# Brevibacillus Laterosporus Bacteremia in an Adult

**DOI:** 10.7759/cureus.10481

**Published:** 2020-09-16

**Authors:** Anna K Curtis, Christian Lamb, Wail M Hassan, John Foxworth

**Affiliations:** 1 Internal Medicine, University of Missouri-Kansas City (Hospital Hills Campus), Kansas City, USA; 2 Internal Medicine, Brooke Army Medical Center, San Antonio, USA; 3 Microbiology, University of Missouri-Kansas City (Hospital Hills Campus), Kansas City, USA

**Keywords:** brevibacillus laterosporus, bacillus, bacteremia, sepsis, bacillus species other than anthracis

## Abstract

*Brevibacillus laterosporus* (*B. laterosporus*) is an aerobic gram-positive bacillus that is rarely associated with human infection. A review of multiple online databases revealed no other cases of bacteremia in an adult involving this organism. Historically, this “canoe-shaped” microbe has been characterized as a pathogen in invertebrates, and information regarding human infection is scarce. We present a clinical vignette of what we believe to be the first reported case of *B. laterosporus* bacteremia in an adult human subject.

## Introduction

*Brevibacillus laterosporus* is an aerobic gram-positive bacillus found in the soil and aquatic environments [1]. This “canoe-shaped” microbe was a member of the *Bacillus* genus until 1996 when a 16s rRNA sequence study showed it to be phylogenetically distinct from the *Bacillus*, *Amphibacillus*, *Paenibacillus* species, and other related organisms [2].

While there are reports of infection in invertebrates, reports of human *B. laterosporus* infection are scarce. Despite an extensive literature search of Google Scholar, Elton B. Stephens Company (EBSCO), PubMed, and Journal Storage (JSTOR) using the key terms “*Brevibacillus*,” “*Bacillus* spp. other than anthracis,” “*Brevibacillus laterosporus*,” and “*Bacillus* bacteremia,” only one case of *B. laterosporus* bacteremia was discovered [3].

The single case of *B. laterosporus* bacteremia (Case A) featured a pediatric patient with medulloblastoma [3]. The patient was receiving long-term chemotherapy for her malignancy via a Hickman catheter when she developed *B. laterosporus* bacteremia which was successfully treated with gentamicin. Months after this infection, she developed a second episode of *B. laterosporus* that was also successfully treated.

Herein, we report the first case of *B. laterosporus* bacteremia in an adult. Discussion of the pathogenicity and management of infections by this organism are also included.

## Case presentation

A 35-year-old African American female with atypical hemolytic uremic syndrome (aHUS) and bilateral nephrectomy receiving intermittent hemodialysis was brought to the emergency department (ED) for vomiting, rigors, and fever that developed during her routine eculizumab infusion at the hospital’s hematology clinic. Her initial blood pressure was 110/96, heart rate was 112 beats/minute, respiratory rate was 24 breaths per minute, with a temperature of 40°C. Physical exam revealed a thin female with a tunneled central venous catheter (CVC) and a mature left brachial hemodialysis fistula. The site of both the tunneled CVC and the fistula were clean, dry, and without erythema. The rest of the physical exam was normal. Laboratory studies showed normocytic anemia near the patient’s documented baseline, without leukocytosis or bandemia. Blood cultures from the tunneled CVC and from the periphery were obtained. The patient was empirically started on vancomycin and cefepime. On hospital day 2, evaluation of the cultures revealed “*Bacillus* spp. other than *anthracis*.” The presence of *B. laterosporus*was confirmed using matrix-assisted laser desorption ionization-time of flight (MALDI-TOF) mass spectrometry (Shimadzu Europa GmbH, Duisburg, Germany). Samples of the organism were sent to a reference laboratory for further testing. Cefepime was discontinued in favor of vancomycin monotherapy.

On the fifth day of hospitalization, antimicrobial zones of inhibition returned from the outside laboratory. Given that no breakpoints are reported in the literature, an interpretation of the zones of inhibition for each antibiotic was inconclusive. Clinicians reviewed the single case report of *B. laterosporus* bacteremia and vancomycin was discontinued in favor of intravenous gentamicin monotherapy.

Repeat blood cultures drawn 48 hours after her presentation were negative for growth by day 7 of hospitalization. The subcutaneous CVC was removed, and a culture of the catheter tip yielded no growth. The patient was continued on gentamicin for 14 days. She improved and remained asymptomatic until discharge. Six months following the initial presentation, the patient reported doing well and continues to receive eculizumab infusions at the hematology clinic without complication.

## Discussion

Human infection by *B. laterosporus* is rare and numerous obstacles exist in the identification and treatment of such infections. Given the lack of reported breakpoints or literature supporting treatment modalities, antibiotic selection against this organism is challenging. Further, hosts of* B. laterosporus* may be immunocompromised and unique microbiologic characteristics of this organism complicate its identification. A review of the single previous case of *B. laterosporus* bacteremia [3] and comparison to the case reported herein can reveal common features of pathogenicity and help guide treatment choices by clinicians.

Several common features exist between our patient and the pediatric patient in Case A [3]. Both individuals were immunocompromised. The patient reported in case A was receiving long term chemotherapy for medulloblastoma. Our patient was receiving eculizumab for aHUS. Additionally, the presence of an indwelling central venous catheter (CVC) was a shared characteristic between these two cases. The patient in case A had a CVC for chemotherapy and our patient had a subcutaneous CVC for dialysis. In both cases, blood cultures obtained from the periphery and from the catheter were positive. The subject in case A presented with an erythematous and tender CVC insertion site, making it reasonable to infer that the catheter site served as a nidus for bacterial growth. In contrast, our subject had no findings suggestive of catheter-related infection. Additionally, subsequent cultures of the catheter tip following its removal were negative.

A review of possible sources of *B. laterosporus* revealed that it is known to be present in the soil, aquatic environments, fermented foods, and probiotic supplements [1]. Our patient was interviewed regarding possible exposures and ultimately denied contact with any of these risk factors. Additionally, clinical pharmacists reviewed local records of patients who had recently received eculizumab and found no other cases of bacteremia following infusion. Unfortunately, attempts to acquire and culture the eculizumab bottle used by our patient were unsuccessful.

Another obstacle when treating *Brevibacillus* spp. infection is antibiotic selection. Currently, there are no reported breakpoints for *Brevibacillus* spp [4]. Breakpoints, in conjunction with the minimum inhibitory concentration (MIC), can be used to determine the sensitivity/resistance patterns of a particular organism in vitro [5].

## Conclusions

*B. laterosporus* is a rare human pathogen. Both reported cases of *B. laterosporus *occurred in immunocompromised hosts with indwelling CVCs. This case reinforces the challenges of treating infections caused by uncommon organisms and attempts to bridge a gap in clinical knowledge regarding this pathogen.
